# Association between subway iron particulate matter exposure and respiratory disease in New York City

**DOI:** 10.1371/journal.pgph.0005335

**Published:** 2026-01-07

**Authors:** Sára Melicharová, Stephenson Strobel, Yongkang Zhang, José A. Pagán, Anqing Hu, Mark Weiner, Masoud Ghandehari

**Affiliations:** 1 Department of Biology, Graduate School of Arts and Science, New York University, New York, New York, United States of America; 2 Division of Health Policy and Economics, Department of Population Health Sciences, Weill Cornell Medical College, New York, New York, United States of America; 3 Department of Family Medicine and Department of Economics, McMaster University, Hamilton, Ontario, Canada; 4 School of Global Public Health, New York University, New York, New York, United States of America; 5 Tandon School of Engineering, New York University, New York, New York, United States of America; 6 Division of Health Informatics, Department of Population Health Sciences, Weill Cornell Medical College, New York, New York, United States of America; The Ohio State University, UNITED STATES OF AMERICA

## Abstract

Particulate matter exposure is linked to increased morbidity and mortality. Iron-rich particulate matter (PM2.5), common in rapid transit systems, is a potential but understudied contributor to respiratory illness. Using electronic health records (EHR) from 452,272 patients in the INSIGHT Clinical Research Network in New York City (2020–2023), we examined whether local iron exposure is associated with asthma, chronic obstructive pulmonary disease (COPD), breathing difficulties, or respiratory inhaler use. Iron exposure was estimated using particulate matter measurements from New York City (NYC) subway stations, linked to each patients residential census block group. To account for potential non-linear relationships, we applied linear probability models and an adjacent block group estimator with paired fixed effects to assess respiratory outcomes across deciles of iron exposure. We found that the relative risk of developing asthma, COPD, or breathing difficulties increased by 6–15% between the lowest two exposure deciles. Beyond this range, there was no significant association between iron exposure and respiratory disease. This suggests that iron exposure from rapid transit is associated with respiratory disease primarily at lower exposure levels, with limited health benefits from marginal reductions in iron exposure at already high exposure levels.

## Introduction

There is growing evidence that exposure to particulate matter (PM) substantially impacts human health. Exposure to air pollution, including fine particulate matter (PM2.5) has been linked to emergency room visits and higher mortality [1,2], adverse infant health outcomes [3–5], and reduced life expectancy [6]. Globally, PM2.5 contributes to millions of premature deaths and adverse perinatal outcomes [7,8]. Localized studies also demonstrate the morbidity burden of PM exposure, with extensive epidemiologic evidence linking PM2.5 to cardiovascular and respiratory illness [9], and elevated mortality among the elderly [10]. Collectively, this evidence suggests that PM2.5 exposure causes significant morbidity and mortality. Mechanistic evidence further supports this link: inhaled PM can generate oxidative stress and inflammatory responses that contribute to respiratory illness. Metal-rich fractions of PM, including iron (Fe), have been shown to drive these biological responses through redox activity and pro-inflammatory signaling [11–13]. While urban PM has historically been dominated by combustion emissions, non-exhaust sources now constitute an increasing proportion of respirable PM. In subway systems, mechanical wear between trains and rails produces especially high concentrations of iron particles [14,15]. These exposures are distinct from combustion-derived PM and may represent an important but understudied environmental health risk.

One understudied source of PM2.5 is from public transportation, which billions of people across the world use every year [16]. Subways account for a large proportion of this public transport and are a locus for unique environmental exposure in iron-rich PM2.5 which is produced by the friction of subway cars traveling along iron tracks. Previous studies of PM and health outcomes are often based on the combustion of fossil fuels and biomass with the evidence on the health effects of exposure to iron-rich PM2.5 lacking. This is concerning because iron exposure has been associated with oxidative stress in vitro [11], poorer health in animal models [12], and increased inflammatory markers in human subjects [13]. Only a few studies have been able to directly link particulate exposure from subways to health outcomes and often only in select populations. For example, a study of Parisian subway workers found that long-term exposure to subway-derived coarse particulate matter (PM10) was associated with increased COPD prevalence and reduced lung function compared to controls, with longer years of work underground linked to greater impairment, although this study did not assess iron specifically [17]. More recent work has highlighted the importance of iron as a dominant component of subway PM. A pilot study in New York City (NYC) found that subway aerosols contained PM2.5 heavily enriched in iron (approximately half of the PM2.5 mass), manganese, and chromium, with concentrations more than 100 times higher than aboveground air. Personal monitoring of subway workers showed exposures ranging from 6 to 469 µg/m³, raising concerns about respiratory and systemic toxicity [18]. Subsequent measurements confirmed that iron constitutes about 43% of PM2.5 mass in the NYC subway, with higher exposures disproportionately affecting low-income and minority commuters [14,19]. Outside of public transportation settings, excessive iron exposure in miners results in lung inflammation and subsequent respiratory diseases [20]. However, it is unclear whether and how much ambient levels of iron-rich PM2.5 exposure, like that found in subways, results in significant medical morbidity [21,22].

NYC provides an important setting for the analysis of this question. The subway system is among the largest in the world, serving approximately 5.5 million daily riders across 472 stations [16,23]. Measurements show that subway stations contain high levels and large variations of iron-rich PM2.5, in some cases exceeding street-level iron concentrations by more than 100-fold [14,24]. On average, iron constitutes about 43% of the total PM2.5 mass in subway stations [14], making it the most significant component compared to other track-level particulate exposures. Given that 55% of NYC adults rely on the subway for commuting [25], a substantial share of the population is routinely exposed to these elevated concentrations. To evaluate the potential health consequences of this exposure, we linked iron-rich PM2.5 measurements from subway platforms to comprehensive electronic health records’ (EHR) data from five major health systems. Leveraging this large dataset, we estimated the effect of subway-related iron exposure on respiratory outcomes. The density of subway stations creates a natural experiment in which nearby communities share similar socioeconomic and environmental conditions but differ in their local subway-related iron exposures. Our results provide insights into how iron exposure of public transportation users may be related to worse health outcomes, which can be used to inform public health interventions to improve population health.

## Materials and methods

### Ethics statement

This study was conducted using de-identified electronic health record data from the INSIGHT Clinical Research Network. Ethics approval was obtained from the Weill Cornell Medicine Institutional Review Board under protocol number 23–04025988, titled “Data-Driven Approach to Identify Determinants of Outcomes and Risk Factors of Cardiovascular Diseases in the New York City Area.” Patient consent was not required, as all data were fully de-identified prior to analysis. Data were accessed for research purposes on July 14, 2023. Authors did not have access to direct identifiers, and all analyses were conducted on de-identified datasets.

### Data and outcomes

We used two data sources to estimate effects of subway related iron exposure on health outcomes. First, we used real-time light scattering-based and gravimetric-based PM2.5 measurements of iron particulate matter collected in nine NYC subway lines, including both on-train and on-platforms. These measurements were collected over two days in October and two days in December of 2021. Subway stations that had particulate matter readings were on the #1, #3, #5, #6, B, C, F, M, and R lines. Our statistical strategy to estimate effects relied on geographic variation in iron exposure across stations and so we focused on the on-platform measurements. We used the highest reading for an observation in the 38 cases where we had multiple exposure readings available (e.g., on a platform in the same station but on different subway lines). Of these 38 stations, using the alternate reading would result in a change in categorization by one decile for 31 of these stations.

Second, we used EHR data from multiple large academic health systems in NYC available through the INSIGHT Clinical Research Network (CRN). This dataset includes over 22 million patient records, with approximately 12 million unique patients. Many patient encounters come from diverse communities across all five boroughs, and the network includes a broad mix of racial/ethnic groups, age spans, and social determinant linkages. INSIGHT showed similar diversity to NYC although with some limitations. In particular, patients who do not access the five academic health systems or rely on safety-net clinics outside participating centers are less well represented. Socioeconomics likely favor those with more consistent access to academic health care. Therefore, while INSIGHT does not perfectly mirror every segment of the NYC population, it provides a large, diverse, and richly characterized sample that is broadly reflective of many areas and subpopulations of the city.

We included only those patients who have an address that is within a NYC census block group and interacted with these five systems during the period of 2020 and 2023. We further excluded anyone living in a NYC census block whose closest stations did not have PM2.5 readings. Our initial sample contained data on 1.78 million individuals in NYC. After our sample restrictions that accounted for our statistical strategy, this included 454,272 patients.

As we expected that iron particulate matter inhalation has a direct effect on respiratory function [11–13], we examined outcomes related to these types of diseases. We examined whether a patient had ever received an International Classification of Diseases, Ninth Revision (ICD-9) or Tenth Revision (ICD-10) diagnosis code of asthma, COPD, or abnormal breathing after an interaction with the health systems in our data. In the last case, these are patients who received an ICD10 R06 or ICD9 786 diagnosis code. As these diagnoses are frequently treated using inhaler medications we examined whether a patient has ever been prescribed two of the more commonly used medications which are ipratropium or salbutamol. Finally, we examined the calendar year in which patients were first diagnosed with COPD or asthma in the EHR.

### Statistical strategy

Our interest was in how local iron-rich particulate exposure is associated with respiratory disease. We assumed that the most consistent daily exposure comes from an individual’s local subway station. This is supported by prior work showing that ambient iron accounts for only 0.34% of street-level PM2.5 in NYC, but on average 43% of subway PM2.5, where it is accompanied by enrichment in other metals including silicon, calcium, sulfur, copper, manganese, and zinc [14]. Because most New Yorkers commute daily by subway, these stations are the main site of iron inhalation exposure [25].

**Fig 1:**
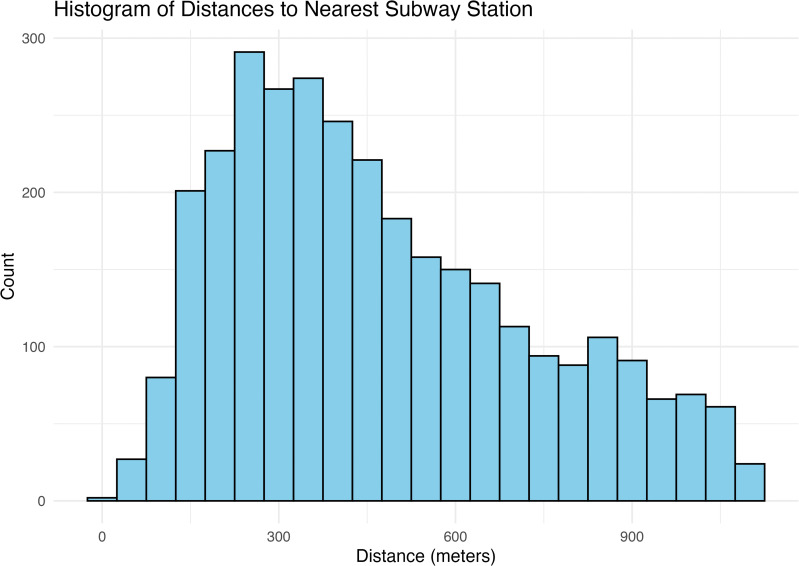
Distribution of distances from census block group centerpoint and nearest subway station in our data.

We geolocated patients to their residential census block group (CBG) and linked each CBG centroid to its nearest subway station. CBGs within NYC are relatively small with the average and median size in our sample being 0.75km and 0.52km squared respectively. In NYC, each CBG is uniquely paired with a single nearest subway station, and no centroid is equidistant between stations. Only ten CBGs have more than one subway station within them. Our analytic sample is restricted to patients who [1] appeared in the EHRs of five academic health systems, [2] had encounters between January 1 2020 and December 31 2023, and [3] lived in CBGs whose nearest station was measured in the subway PM2.5 dataset. This yields 452,272 unique patients across 3,180 CBGs (out of 6,587 total in NYC). Stations are densely distributed: the median centroid of a CBG in our sample lies within 500 meters of a subway station, and 95% within 1,000 meters (Fig 1).

We estimated models with two variables of interest. First, to mimic previous studies of iron PM, we used the direct measure of iron-rich PM2.5 of a local subway station. These regressions are specified as

where i indexes individuals, j their locus CBG, and p the ordered pair of a locus CBG and an adjacent CBG with a different station. $\theta_{p}$ is a CBG pair fixed effect which is discussed in more detail below. However, in our main regressions of interest, we used a variation of this variable of interest. To estimate whether there are non-linear effects of iron-rich PM2.5 on these outcomes, we created deciles of iron exposure and used these fixed effects in our regressions.

**Fig 2:**
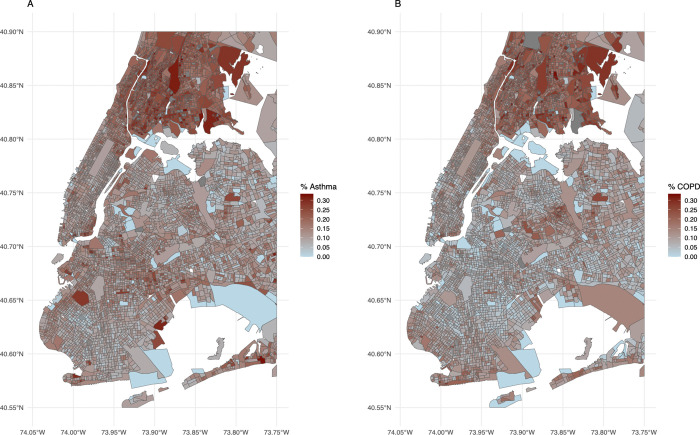
Proportion of persons with asthma and COPD in INSIGHT data by census block group. (Base map shapefiles from the U.S. Census Bureau TIGER/Line. https://www.census.gov/geographies/mapping-files/time-series/geo/tiger-line-file.2021.html).

**Fig 3:**
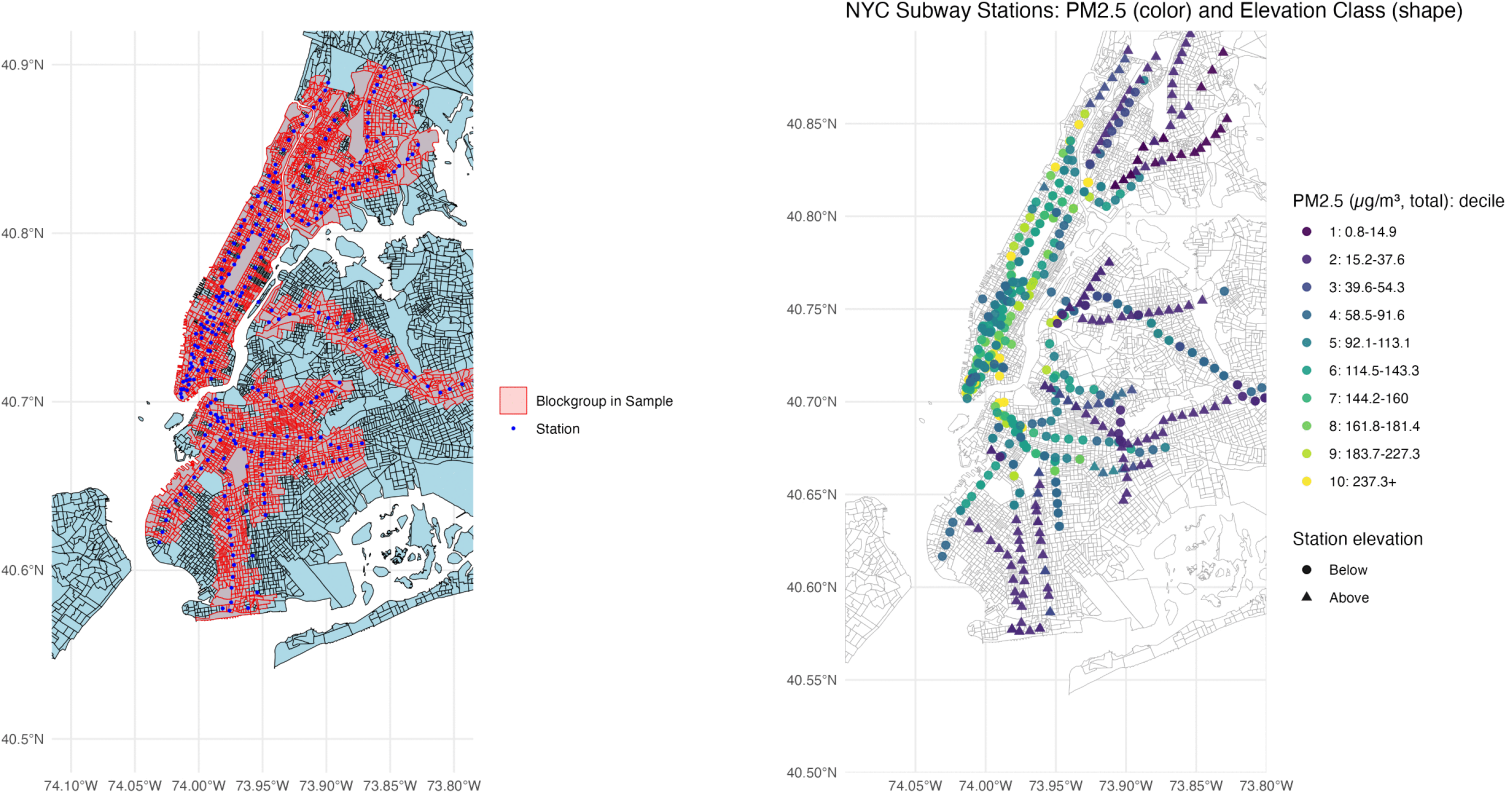
Locations in New York City included in sample and by decile of iron exposure from their local subway station. These census block groups are adjacent to one another but have different subway stations that are closest to the block group center point. (Base map shapefiles from the U.S. Census Bureau TIGER/Line. https://www.census.gov/geographies/mapping-files/time-series/geo/tiger-line-file.2021.html).

A simple approach to try and estimate the association between subway iron levels and respiratory disease would be to regress respiratory outcomes on iron levels at an individual’s nearest station. However, station design and location strongly correlate with socioeconomic context. For example, Bronx stations which are mostly above ground, have low iron levels but higher COPD and asthma rates in socioeconomically disadvantaged areas, while deep underground Manhattan stations have high iron levels but lower respiratory disease rates in wealthier communities. Prior work (e.g., Azad et al.) has also shown socioeconomic status and iron-rich PM 2.5 exposure correlations across NYC. Figs 2 and 3 illustrate these patterns, suggesting potential bias if unobserved socioeconomic conditions are not adequately controlled.

**Fig 4:**
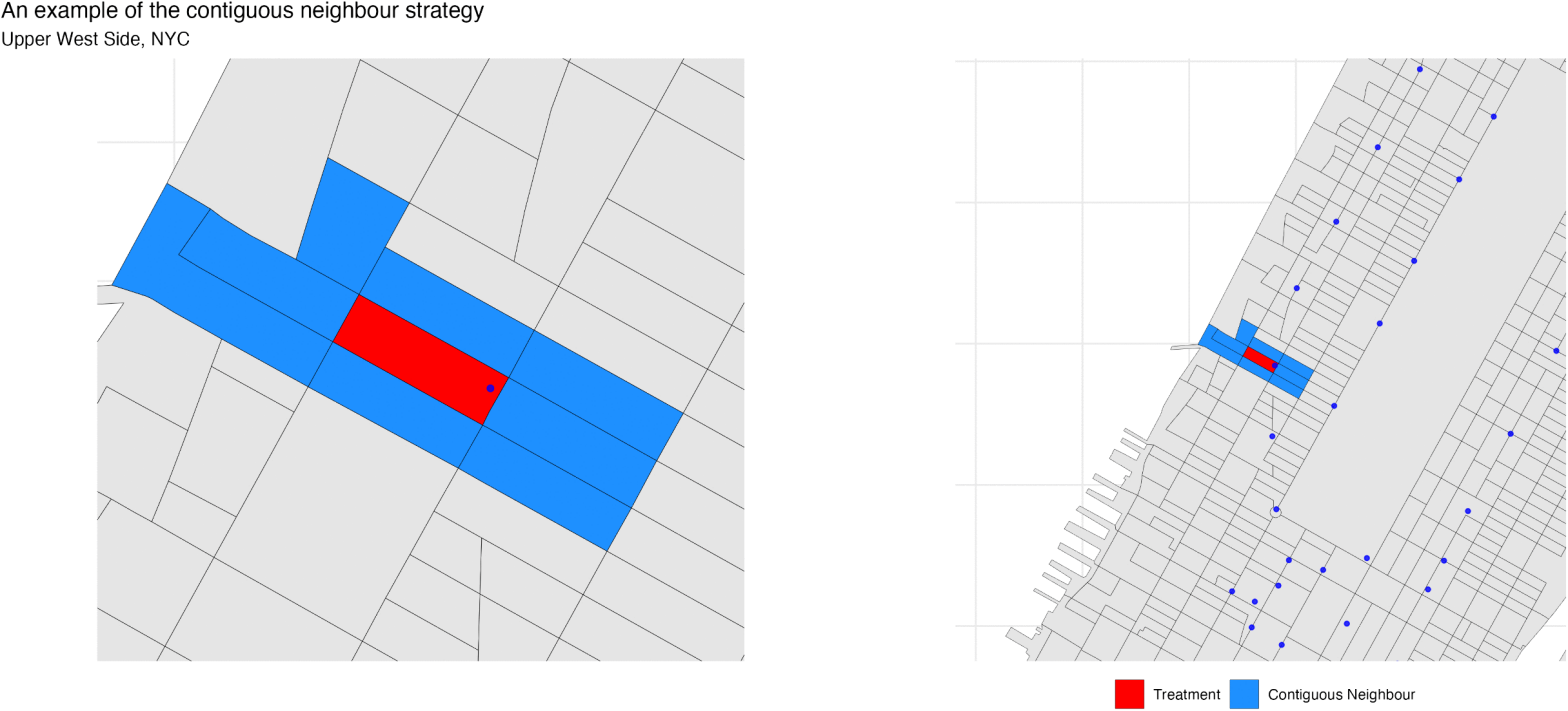
An example of the contiguous block strategy. Persons living in the red census block group are compared to persons living in adjacent block groups with different subway stations. There are five potential comparison groups in the case of this census block group in the Upper West side of NYC. (Base map shapefiles from the U.S. Census Bureau TIGER/Line. https://www.census.gov/geographies/mapping-files/time-series/geo/tiger-line-file.2021.html).

To mitigate this bias, we compared patients in one CBG to patients in adjacent CBGs with different nearest subway stations [26,27]. Because NYC CBGs are small and densely populated, these comparisons often involve neighbors living only a few hundred meters apart, reducing unmeasured socioeconomic and environmental confounding. Fig 4 illustrates this strategy for a block on the Upper West Side, where residents of the red central block are compared to residents of five surrounding blue blocks.

This design means individuals can appear multiple times if their block borders several comparators. For example, a resident of the central block in Fig 4 could enter regressions five times, once for each neighbor. As a result, our 452,272 patients expands to 2.6 million observations. Each block–neighbor pair is assigned its own fixed effect, ensuring comparisons are identified only by within-pair differences in iron exposure. More concretely, our regression specification is:


$$y_{ip}=\sum_{\text{2}}^{\text{1}\text{0}}\beta_{d}I\;\left({IronDecile}_{j}=d\right)+\theta_{p}+\in_{ip}$$

where i indexes individuals, j their locus CBG, and p the ordered pair of a locus CBG and an adjacent CBG with a different station. *IronDecile* bins subway iron readings into deciles (see legend of Fig 2), with decile 1 as the reference, and $\theta_{p}$ as the CBG pair fixed-effect. We are interested in the effect of iron-rich PM2.5 on an outcome y which is the development of respiratory disease or use of a respiratory inhaler medication. This same strategy also allowed us to test whether there are systematic differences in adjacent CBGs we are comparing. We substituted y for a battery of demographic and health variables to test whether iron decile is associated with these outcomes. If this adjacent CBG strategy minimizes socioeconomic and demographic differences, we should expect to see minimal associations between y and our iron deciles.

We report results without these pair fixed effects, but our preferred model includes them. Because individuals can appear multiple times across pairs, this induces mechanical correlation in residuals. To correct for this, we clustered standard errors at the locus CBG level [28]. We used a significance threshold of 1% for our hypothesis testing.

## Results

Fig 3 maps the distribution of stations through NYC by our variable of interest, the PM2.5 of iron. These data are highly left skewed with most census block groups near to stations with lower iron particulate readings. However, there are some census block groups proximate to stations with very high iron exposure of over 500 μg/m³ PM2.5 particulate matter readings. In total, 55% or 1765 of our CBGs are located near stations where iron-rich PM2.5 is above 55ppm.

Panel A of Table 1 reports the demographics of the sample population in the INSIGHT health data. Our sample is 38% white and 18.5% Hispanic. The sample is 41.2% male. We provide the regression estimates of these demographic outcomes on our iron-rich PM2.5 measure while controlling for our block group pair fixed effects as a check on whether our strategy effectively controls for observable confounding. These are demonstrated in panel B of Table 1. We find some statistically significant differences especially on race/ethnicity outcomes and whether or not a patient has been diagnosed with hypertension; however, these differences are clinically very small. For example, our estimates suggest that after controlling for our contiguous pair fixed effects, moving from the 10th to 90th percentile of local station iron inhalation increases the absolute probability of being white by approximately 1.98%. Similarly, it increases the absolute probability of a hypertension diagnosis by 0.85%. This suggests very clinically small levels of demographic and health selection within our comparator census block group pairs.

**Table 1:** Summary Statistics and Regression Results using these demographics as outcome variables. Panel A describes the sample of individuals in our data. Panel B are results from a linear regression where the variable is regressed on iron outcomes and CBG fixed effects. The estimate is the association between iron exposure and the outcome which is effectively a test on whether there are clinically meaningful differences between adjacent blocks. * indicates that results are statistically significant at the 5% level.

Panel A: Summary Statistics		
Variable	Mean	SD
Male (proportion)	0.412	0.492
Year of Birth	1969.2	19.77
White (proportion)	0.38	0.485
African American (proportion)	0.119	0.324
Asian (proportion)	0.056	0.23
Hispanic (proportion)	0.185	0.388
Has Hypertension (proportion)	0.360	0.480
Panel B: Regression Results		
Outcome	Estimated effect of moving from 10th to 90th percentile of iron PM2.5	SE
Male	0.0015	0.0029
Year of Birth	0.3080	0.2300
White	0.0198*	0.0058
Black	0.0221*	0.0034
Asian	0.0059*	0.0022
Hispanic	-0.0006	0.0060
Has Hypertension	-0.0085*	0.0032

Table 2 demonstrates the estimates of our regressions with a linear PM2.5 outcome. Regression results without block group fixed effects counterintuitively suggest that increasing local subway PM2.5 by 100μg/m³ is associated with reductions in the probability of developing respiratory related diseases such as asthma or COPD by 1.5% each. This is suggestive evidence that unobserved variables are driving these associations. Our results that control for block level fixed effects display similarly negative, but far smaller results; we find, for example, that increasing local subway station iron-rich PM2.5 by 200 reduces the probability of asthma by 0.3% although this is not statistically significant. Increasing local subway station iron-rich PM2.5 by 200 μg/m³ reduces the probability of breathing difficulties by 0.7%. Ventolin and atrovent prescriptions and year of diagnosis are similarly counterintuitively associated with local subway iron inhalation; higher levels of iron are associated with fewer prescriptions and a later diagnosis.

**Table 2:** Regression results of outcomes on a linear value of the local subway station iron-rich PM2.5. Regressions with pair fixed effects use our contiguous block group estimators whereas those without are naive regressions.

Panel A: Effects on asthma, COPD, and abnormal breathing diagnoses				
Outcome	Estimate	SE	Pair fixed effects	95% CI
**Asthma**	-0.000156**	**5E-05**	**N**	**[-0.000254, -0.000058]**
**Asthma**	-0.000015	1E-05	Y	[-0.000035, 0.000005]
**COPD**	-0.000158***	4.4E-05	N	[-0.000244, -0.000072]
**COPD**	-0.000027**	9E-06	Y	[-0.000045, -0.000009]
**Abnormal Breathing**	-0.000135	7.2E-05	N	[-0.000277, 0.000007]
**Abnormal Breathing**	-0.000036*	1.4E-05	Y	[-0.000064, -0.000008]
**Year Dx Asthma**	0.003946***	0.001117	N	[0.001756, 0.006136]
**Year Dx Asthma**	0.000347*	0.000171	Y	[0.000013, 0.000681]
**Year Dx COPD**	0.004077***	0.001001	N	[0.002115, 0.006039]
**Year Dx COPD**	-0.000134	0.000217	Y	[-0.000559, 0.000291]
**Asthma**	-0.000156**	5E-05	N	[-0.000254, -0.000058]
**Panel B: Effects on Inhaler Prescriptions**				
**Outcome**	**Estimate**	**SE**	**Pair Fixed Effects**	**95% CI**
**Ever Ventolin**	0.000131***	1.3E-05	N	[0.000106, 0.000156]
**Ever Ventolin**	-0.000019**	7E-06	Y	[-0.000033, -0.000005]
**Ever Atrovent**	-0.000019**	7E-06	N	[-0.000033, -0.000005]
**Ever Atrovent**	-0.000005*	2E-06	Y	[-0.000009, -0.000001]
**Ventolin**	0.000444***	7.6E-05	N	[0.000295, 0.000593]
**Ventolin**	-0.000155***	3.9E-05	Y	[-0.000232, -0.000078]
**Atrovent**	-0.000238***	6.4E-05	N	[-0.000364, -0.000112]
**Atrovent**	-0.000096*	4E-05	Y	[-0.000174, -0.000018]

**Fig 5:**
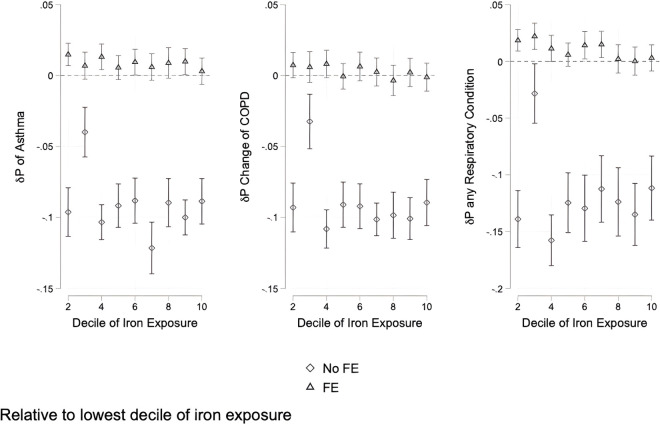
Effects of decile of iron exposure on probability of an asthma, COPD or breathing abnormality diagnosis. Effects are relative to the lowest decile.

These counterintuitive linear results are explained in our non-linear regressions where we bin local subway iron-rich PM2.5 estimates into deciles and include our paired fixed effects. We find consistent results across disease outcomes (Fig 5). Between deciles two and ten of iron-rich PM2.5 exposure, its relationship with developing respiratory diseases is essentially flat. However, between decile one and these remaining deciles there is a 1.5, 1 and 2.5% increase in the probability of developing asthma, COPD, and breathing difficulty respectively. On a baseline of approximately 15% prevalence in our sample, this means an increase in the relative risk of these diseases by 6.6% to 16%.

**Fig 6:**
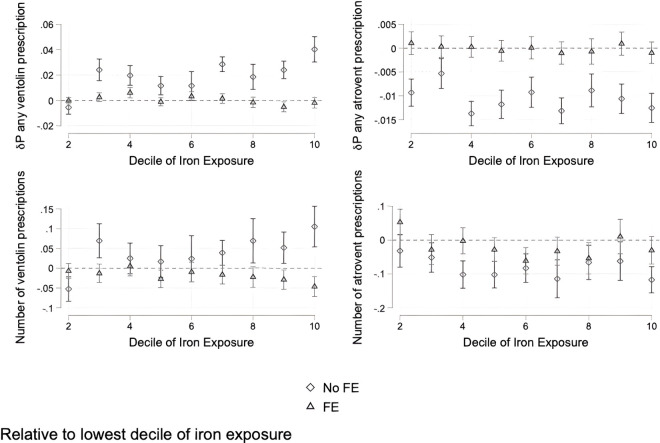
Effects of decile of iron exposure on probability of receiving an inhaler medication prescription and number of prescriptions. Effects are relative to the lowest decile.

We find much more muted results in our medication outcomes (Fig 6). There is no consistent relationship between iron-rich PM2.5 decile and whether a patient has ever received any prescription for atrovent or ventolin. We find similar results for the number of prescriptions of these medications a patient receives.

**Fig 7:**
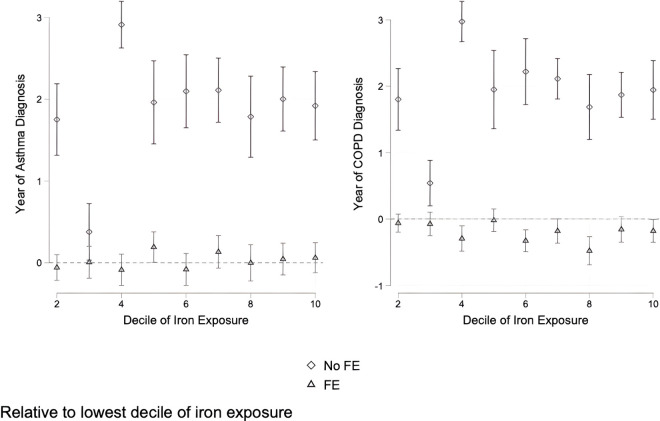
Effects of decile of iron exposure on year of first diagnosis for asthma or COPD. Effects are relative to the lowest decile.

However, among people with a COPD, asthma, or breathing difficulty diagnosis, we find some evidence that at higher deciles of local subway iron exposure, there are reductions in the first year of diagnosis (Fig 7). These negative relationships almost exclusively occur in earlier COPD diagnosis. Being in the 8th decile of iron exposure for example, reduces the year of first diagnosis by about 5 months.

## Discussion

We demonstrate three important results on the association between local subway iron exposure and development of respiratory disease. First, we show that estimating linear relationships between our outcome variables and local subway PM2.5 iron content are inconsistent and often provide contradictory and counterintuitive results. These regressions suggest that higher iron-rich PM2.5 at an individual’s local subway station in NYC is associated with lower respiratory disease. Second, when we employed non-linear models that use decile of iron exposure as a variable of interest we found results consistent with increased iron exposure resulting in worse health. These suggest that increasing PM2.5 iron content in subways correlates with a higher probability of developing respiratory diseases and, in the case of COPD, earlier diagnosis. Finally, marginal increases in this relationship only occur at low levels of iron exposure. This is to say that higher exposure still correlates with higher respiratory disease relative to exposure in the first decile, but that this relationship plateaus after moving from the first to second decile of exposure. An increased probability of these diseases occurs at relatively low levels in local subway station iron content but then remains stable at a higher probability.

Our first results may explain the inconsistent results seen in the existing iron exposure literature. For example prior studies in individuals exposed to iron particulate matter in mines demonstrate positive [29,30], and null relationships [31] with lung disease. These mining studies tend to compare individuals with mining exposure to those without and do not account for a dose response. Similarly, the Parisian subway worker study reported that long-term exposure to subway-derived coarse PM10 was associated with increased COPD prevalence and reduced lung function, although the study did not characterize specific particle composition [17]. In contrast, the Grass et al. study found that subway aerosols were heavily enriched in iron, manganese, and chromium, with iron comprising nearly half of the PM2.5 mass, raising concerns about respiratory toxicity [18]. These studies are limited by their modelling choices. In the case of the Paris study a logit model was used which necessarily constrains estimates to a single effect rather than examine a dose response. The Grass et. al. study uses a similar exposed vs. non-exposed set up as the previous mining studies. These studies also suggest both an epidemiologic association with adverse respiratory outcomes and a mechanistic basis for concern but were limited by focusing on worker populations or small-scale environmental measurements, without adjustment for neighborhood-level socioeconomic or environmental confounding. We essentially replicated these issues across outcome variables in our regressions without block group fixed effects where we found that increasing local subway iron content can be associated with decreasing probability of COPD, breathing issues, and asthma.

Our non-linear estimates help explain the counterintuitive findings from the linear regressions. The pattern reflects a relatively flat relationship across higher deciles of PM2.5 iron exposure, but when compared to the very lowest decile, there is a consistent increase in the probability of developing respiratory illness. This suggests that reducing exposure to particulate iron in PM2.5 could meaningfully lower the incidence of respiratory disease.

However, the non-linear regressions show where mitigation measures may have the most impact and it is not the case that mitigation improves outcomes across all levels of exposure. We only observe marginal changes in respiratory outcomes when moving from the lowest decile to the second lowest decile of iron exposure. Moving from the second decile to any other decile of iron exposure does not increase the probability of developing respiratory diseases. This suggests that iron mitigation measures that move exposure into this lowest decile will reduce respiratory diseases. Mitigation measures that move patients at higher deciles, for example, from the fifth to the fourth decile, will have limited impact on respiratory outcomes. This result is seen in other studies on pollution exposure [2]. While it is difficult to estimate the exposure to iron-rich PM2.5 on the New York subway, our results suggest that modest levels of iron inhalation exposure may increase the risk of developing respiratory disease. The potential biological mechanisms underlying these associations likely involve the redox activity of iron-rich particles. Iron can catalyze Fenton-type reactions that generate reactive oxygen species, leading to oxidative stress and cellular damage. This process has been demonstrated in vitro [11], in animal models [12], and in biomarker studies of subway workers [18]. In parallel, oxidative stress activates inflammatory signaling cascades, resulting in airway inflammation that contributes to respiratory disease [13]. Chronic or repeated exposures to iron-rich PM may therefore accelerate airway injury, providing a plausible biological basis for the respiratory associations observed in our analysis.

## Limitations

Our results are subject to several limitations. We acknowledge the possibility of other unobserved and uncontrolled variables that may be driving our observed relationship between subway station iron exposure and respiratory illness. Related to this point, because we are reliant on subway iron exposure data collected at one point in time, we assume that this is the routine exposure that individuals receive at these stations. Iron exposure within a station may change during different seasons, at different temperatures, and with different foot traffic [14,24]. This may not occur in a systematic way across subway stations. Similarly, while subway use within New York City is widely prevalent, we do not observe an individual’s actual subway use. We do not know how often a person is in the subway, or whether the most proximate station to an individual is a person’s usual way of accessing the Metropolitan Transportation Authority (MTA). Our data also do not contain information on where patients work, how long they have lived at their recorded address, or whether exposures may have occurred at a previous residence. We are therefore unable to account for occupational exposures or residential mobility, which may attenuate the precision of our exposure assignments. However, our use of block-group fixed effects partially mitigates this concern by ensuring comparisons are made between geographically proximate populations with similar underlying characteristics. The goal of our statistical strategy is to remove this issue as individuals living within several blocks of one another should, in aggregate, be similar in their sociodemographics and in their subway use. New York is also a particularly unique city, in its wealth, demographics, and use of public transit. It may not generalize to other contexts or other cities.

Another major limitation is the interpretability of our estimates. Our results are best interpreted as giving a sign to the directionality of the relationship between iron exposure and probability of respiratory disease. As we do not directly observe the total iron dose that an individual receives over their lifetime from subway exposure, our assumption is that more local exposure results in more iron inhalation. Our non-linear results suggest that this relationship is not constant, but we also cannot estimate the specific exposure where this bend in the relationship occurs. However, prior studies have reported that subway PM2.5 concentrations in NYC range from ~35–200 μg/m³ [24], with iron comprising about 43% of the mass [14].These findings suggest that significant health concerns may arise at exposure levels comparable to those measured in the NYC subway system, though we cannot estimate a precise threshold.

Finally, our outcomes of interest may be subject to measurement error. We find that the prevalence of coded diagnoses for asthma and COPD in our dataset is approximately 15%. This reflects the clinical population captured by the INSIGHT network and does not necessarily represent the true prevalence in the general NYC population. Reported prevalence of asthma in NYC is typically around 10–11% among adults and higher among children, while COPD prevalence is generally lower, around 5–7% among adults, according to city and national surveillance data [32–36]. The higher prevalence we observe likely reflects both the clinical population in our dataset and the greater burden of chronic respiratory disease in NYC compared to national averages [37].

## Conclusion

We demonstrate an association between iron particulate matter in NYC subways and the probability of being diagnosed with respiratory conditions. Our findings suggest that increasing iron particulate exposure is associated with increased probability of respiratory diseases and earlier diagnoses. These results shed light on whether iron exposure should be considered a serious environmental hazard. However, we also demonstrate that this relationship rapidly plateaus. At lower deciles of iron exposure there are increases in probability of respiratory diagnoses that are stable at higher deciles. This suggests that iron exposure may be associated with respiratory illness at levels much lower than those currently considered safe but, once this threshold is crossed, additional exposure does not substantially worsen outcomes.
